# Recent increase of breast cancer incidence among women under the age of forty

**DOI:** 10.1038/sj.bjc.6603783

**Published:** 2007-05-29

**Authors:** C Bouchardy, G Fioretta, H M Verkooijen, G Vlastos, P Schaefer, J-F Delaloye, I Neyroud-Caspar, S Balmer Majno, Y Wespi, M Forni, P Chappuis, A-P Sappino, E Rapiti

**Affiliations:** 1 Geneva Cancer Registry, Institute for Social and Preventive Medicine, University of Geneva, 55, bd de la Cluse, 1205 Geneva, Switzerland; 2 Department of Community, Occupational and Family Medicine, National University of Singapore, 16 Medical Drive, 117597, Singapore; 3 Senology and Surgical Gynecologic Oncology Unit, Department of Gynecology and Obstetrics, Geneva University Hospitals, Bd de la Cluse 32, 1205 Geneva, Switzerland; 4 Department of Gynecology and Obstetrics, Vaud University Hospitals, CHUV, Rue du Bugnon 21, 1005 Lausanne, Switzerland; 5 Division of Radio-Oncology, Geneva University Hospitals, Av. de la Roseraie 53, 1205 Geneva, Switzerland; 6 Association of Physicians of the canton of Geneva, Rue Micheli-du-Crest 12, 1205 Geneva, Switzerland; 7 Division of Oncology, Department of Medicine, Geneva University Hospitals, Rue Micheli-du-Crest 24, 1211Geneva 14, Switzerland

**Keywords:** breast cancer, young patients, trend, incidence rates, cancer registry, population-based study

## Abstract

Using data from the Geneva Cancer Registry, we found that in 2002–2004, breast cancer incidence in women aged 25–39 years increased by 46.7% per year (95% CI: 7.1–74.0, *P*=0.015), which surveillance or detection bias may not fully explain.

Health professionals and patient support groups in Geneva, Switzerland, have recently expressed concern about an apparently increasing number of very young breast cancer patients. Owing to the average 2-year delay in cancer registration, we have only now been able to investigate this observation using incidence data up to the year 2004 among women residents in Geneva.

## MATERIALS AND METHODS

The Geneva cancer registry, functional since 1970 and covering the whole population of the canton (approximately 435 000 inhabitants), is considered comprehensive with a low percentage (<2%) of cases recorded from death certificates only (Bouchardy, 1997). Trained staff systematically abstract data from reports of all pathology laboratories and public hospitals. Private practitioners regularly fill out questionnaires to complete missing clinical and therapeutic data; death certificates are systematically consulted.

We included all incident invasive breast cancer cases diagnosed in the resident population of the canton between 1995 and 2004 (*n*=3608), with the population at risk considered as the resident population at the middle of each relevant year, obtained from the Cantonal Population Office. We calculated annual incidence rates for five age groups: 25–39, 40–49, 50–69, 70–79, and ⩾80 years. Trends in age-specific annual incidence rates were calculated by log-linear Poisson regression implemented in the generalised linear interactive modelling statistical package (Francis *et al*, 1993). We calculated mean annual rates of increase over the whole period to test for a continuous progressive increase and calculated the mean annual rates for the last 3-year period only to test for a recent, sudden increase. For women aged 25–39 years, we compared patient and tumour characteristics before and when the increase occurred by *χ*^2^ test for heterogeneity.

Variables of interest included family history of breast or ovarian cancer (positive if one first-degree or two second-degree relatives were affected, and negative), method of detection (mammography or clinical screening, breast self-examination, symptoms or other), modalities of diagnostic assessment (clinical status, mammography or ultrasound, magnetic resonance imaging (MRI): yes *vs* no), histological type (ductal: ICD-O code 8500, lobular: ICD-0 code 8520 and 8522, and other), differentiation (grades I–III, and unknown coded according to the International Classification of Diseases for Oncology, ICD-O (ICD-O International Classification of Diseases for Oncology, 1976)), oestrogen and progesterone receptor status (positive (if ⩾10% of cells expressed receptors), negative, and unknown), mean pathological tumour size (in mm), and stage (coded according to the tumour, node and metastasis TNM classification (TNM Classification of malignant tumours, 1992)). We used the pathologic pTNM classification system or, when absent, the clinical cTNM classification. Tumour stage was considered as stage I (T1 and N0), stage II (T0 or T1 and N1, T2 and N0 or N1, T3 and N0), stage III (T0 or T1 or T2 and N2, T3 and N1 or N2, T4 and any N, any T and N3), stage IV (M1) and unknown. We also examined the proportion of tumours with clinical T0 N0 M0.

## RESULTS

**Figure 1:**
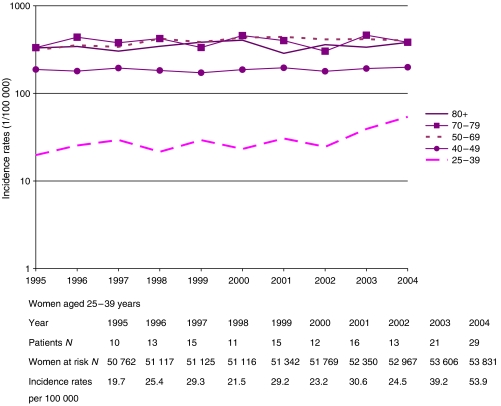
Trends(logarithmic scale) in annual breast cancer incidence rates by age group in Geneva, Switzerland.

For women aged 25–39 years, breast cancer rates were steady until 2002 and increased sharply thereafter, being 19.7 per 100 000 in 1995, rising to 53.9 per 100 000 in 2004 (Figure 1). Table 1 presents results of trend tests for the period 1995–2004 (middle columns) and for the last 3-year period 2002–2004 (right column). The mean annual increase was 8.7% (95% CI: 2.8–15.0, *P*=0.003) over the whole period. The entire increase occurred in the last 3-year period (2002–2004) with a mean annual increase of 46.7% (95% CI: 7.1–74.0%, *P*=0.015). Cancers at ages 25–39 represented 3.4% of all breast cancers in 1995 and 7.2% in 2004 (*P*=0.032). Also, since 1970 (when cancer registration started in Geneva) no such increase has been observed (data not shown). For the other age groups, incidence remained fairly stable, except for women aged 50–69 years among whom it increased at an average of 2.6% per year, from 1998 to 2002, when it stabilised (Table 1, Figure 1).

Table 2 shows tumour characteristics among women aged 25–39 years in 1995–2001 and 2002–2004. We found a significant increase in diagnoses diagnosed by MRI. In particular, 26% of breast cancers in young women were diagnosed by MRI in 1995–2001 compared with 48% in 2002–2004 (*P*=0.006). Screen-detected cancers increased non-significantly from 9 to 19%. The proportion of stage I cancers slightly decreased from 40 to 33%, whereas the proportion of stage II cancers remained relatively constant around 45%. Only three women had non-clinical T0 N0 tumours, one in the first and two in the second period. The mean tumour size remained unchanged over the whole study period, at 21 mm in 1995–2001 and 20 mm in 2002–2004 (*P*=0.817). We observed no increase in the proportion of young patients with a positive familial history of breast cancer.

## DISCUSSION

In Geneva, breast cancer incidence in women aged <40 years has recently doubled. This increase may be partly explained by a higher screening frequency of younger women and better surveillance and recognition of familial risk factors. Improved tumour detection through advances in imaging techniques may also be involved, since in 1995–2001 less than 30% of breast cancer diagnoses in young women involved MRI compared with nearly 50% in 2002–2004 (*P*=0.006).

Nevertheless, these detection biases seem unlikely to explain fully the observed incidence increase. Screening and improvement in diagnostic techniques should lead to a shift in stage distribution towards earlier stages, whereas in our population, this did not change significantly, and the proportion of clinically palpable tumours remained constant. The screening programme implemented in Geneva 10 years ago targets women aged 50–69 years, and screening for breast cancer in women <40 years is rare (Lutz *et al*, 2000). Furthermore, the proportion of patients reporting a positive family history has remained relatively stable around 30% between the two periods. We can also reasonably rule out an increase in fortuitous discovery of contralateral breast cancer as only two women were diagnosed with synchronous breast cancer in 2002 and 2003 respectively.

Although significant, our observation is based on only 63 patients diagnosed in 2002–2004, and should be interpreted with caution. With respect to any change in population estimates, the young resident female population grew smoothly over the study period, with no sudden increase in 2002–2004 (Figure 1). Among other cancer sites of women aged 25–39 years, we found a significant increase of melanoma (mean annual increase of 6.9%; 95% CI: 0.3–13.8, *P*=0.038) starting early in the study period, but this is already a well-documented phenomenon in Switzerland (Association of Swiss Cancer Registries (ASCR), 2002).

Other reasons should be explored to explain this increase in breast cancer incidence in younger women. Most known breast cancer risk factors, including nulliparity or later age at first full-term pregnancy, early menarche, dietary habits, alcohol intake, and lack of physical activity, apply at all ages. However, some other factors, such as family history of breast and/or ovarian cancer, *in utero* exposure, oral contraceptive use, smoking, and breast radiation are more relevant to young women (Yankaskas, 2005; Colditz *et al*, 2006).

In Geneva, prevalence of obesity has increased among adolescents and young females (Morabia and Costanza, 2005), but, in contrast to post-menopausal women, it appears to be protective against breast cancer in young women (Bouchardy *et al*, 1990; Magnusson *et al*, 2005; Michels *et al*, 2006). Smoking still seems to be increasing among women in Geneva and, compared with older women, young women begin smoking cigarettes at a much earlier age and are heavier smokers (Costanza *et al*, 2006). No causal relation between breast cancer and smoking is generally accepted although any impact could be greater on young women (Miller *et al*, 2007). In this study, we can exclude an association with previous cancer treatment, given that only one woman was so affected (bilateral ovarian cancer).

In comparison, for Europe and the United States, we found no recent or sudden increase in breast cancer incidence among women under 40 years in the two main public-use cancer registry data sets, the Surveillance Epidemiology and End Results (SEER) and Cancer Incidence in Five Continents (CIF) (International Agency for Research on Cancer (IARC), 2002; National Cancer Institute (NCI), 2004). However, the latest available years were 1997 for CIF and 2003 for SEER.

In conclusion, we observed a significant increase in young breast cancer patients. At present, we cannot definitively rule out an increased surveillance and detection bias and we cannot confidently conclude a sustained increase. Careful surveillance of recent trends of breast cancer incidence is required for young women. If other population-based cancer registries confirm this trend, further research on breast cancer risk factors, including any acting *in utero* and early in life, would be indicated.

## Figures and Tables

**Table 1:** Mean annual increase of breast cancer incidence rates by age group for the whole study period and for the last 3-year period and *P*-value for trend tests

	**Incidence rates per 100 000**		**Trend test for the whole study period 1995–2004^a^**			**Trend test for the last 3-year period of the study 2002–2004^b^**		
**Age**	**1995**	**2004**	**Mean annual increase (95% CI)**		****P**** **-value**	**Mean annual increase (95% CI)**		****P**-value**
25–39	19.7	53.9	+8.7	(2.8–15.0)	0.003	+46.7	(7.1–100.8)	0.015
40–49	187.3	198.8	−0.6	(−2.8–3.4)	0.700	+5.4	(−11.6–27.7)	0.555
50–69	312.4	400.2	+2.6	(1.0–4.2)	0.001	−1.7	(−11.7–8.3)	0.737
70–79	332.6	383.5	−0.4	(−2.3–3.3)	0.756	+11.0	(−7.1–32.6)	0.247
⩾80	330.7	381.4	+0.9	(−2.5–4.5)	0.590	+3.1	(−16.9–27.8)	0.781

CI=confidence interval.

a Poisson regression testing trends of annual incidence rates during the period 1995–2004.

b Poisson regression testing trends of annual incidence rates during the period 2002–2004.

**Table 2:** Comparison of tumour assessment and characteristics of breast cancer patients aged <40 years before and after the increase began

	**1995–2001**		**2002–2004**		
**Patient and tumour characteristics**	**Number**	**(%)**	**Number**	**(%)**	****P**-value for heterogeneity test**
*Age at diagnosis*					
25–29 years	6	(7)	6	(10)	0.523
30–34 years	32	(35)	17	(27)	
35–39 years	54	(59)	40	(64)	
*Method of detection*					
Screening	8	(9)	12	(19)	0.168
Breast self-examination	66	(72)	40	(64)	
Symptoms, other	18	(20)	11	(18)	
*Diagnostic assessment*					
Clinical status^a^	81	(88)	58	(92)	0.419
Mammography or ultrasound^a^	80	(87)	56	(88)	0.719
MRI^a^	24	(26)	30	(48)	0.006
*Family history*					
Negative	64	(70)	41	(65)	0.557
Positive	28	(30)	22	(35)	
*Stage*					
I	37	(40)	21	(33)	0.673
II	41	(45)	27	(43)	
III	7	(8)	9	(14)	
IV	4	(4)	3	(5)	
Unknown	3	(3)	3	(5)	
*Histology*					
Ductal	81	(88)	60	(95)	0.078
Lobular	0	(0)	1	(2)	
Other	11	(12)	2	(3)	
*Grade*					
I	15	(16)	8	(13)	0.217
II	33	(36)	33	(52)	
III	38	(41)	20	(32)	
Unknown	6	(7)	2	(3)	
*Oestrogen receptor status*					
Negative	33	(36)	18	(29)	0.630
Positive	56	(61)	43	(68)	
Unknown	3	(3)	2	(3)	
*Progesterone receptor status*					
Negative	37	(40)	25	(40)	0.997
Positive	52	(57)	36	(57)	
Unknown	3	(3)	2	(3)	
Total	92	(100)	63	(100)	

MRI=magnetic resonance imaging.

a Binary variables classified as yes *vs* no.
